# The role of the *Chitinase 3-Like 1 (CHI3L1) genes* in the preeclampsia pathophysiology

**DOI:** 10.1590/1806-9282.20231574

**Published:** 2024-07-19

**Authors:** Nigar Mammadova, Sibel Özler, Belma Gözde Özdemir, Fazıl Avcı, Nadir Koçak, Ersin Çintesun, Gökçen Örgül, Çetin Çelik

**Affiliations:** 1 Afiyet Hospital, Private Clinic of Obstetrics and Gynecology – İstanbul, Turkey.; 2 Selcuk University, Faculty of Medicine, Department of Obstetrics and Gynecology – Konya, Turkey.; 3 Selcuk University, Faculty of Medicine, Department of Medical Genetics – Konya, Turkey.

**Keywords:** Chitinase-3-Like Protein 1, Etiology, Preeclampsia

## Abstract

**OBJECTIVE:**

The aim of this study was to investigate the relationship between *Chitinase 3-Like 1 gene* polymorphisms and the occurrence of preeclampsia in a selected cohort of pregnant women.

**METHODS:**

A total of 75 pregnant women participated in the study, 35 of whom were diagnosed with preeclampsia, while 40 served as healthy controls. The preeclamptic group was subdivided based on severity. Real-time polymerase chain reaction was employed to analyze the serum samples for variations in *Chitinase 3-Like 1 gene* polymorphisms.

**RESULTS:**

The rs880633 polymorphism was found to be significantly more frequent in the control group (80%) compared with the overall preeclamptic group (60%) (p<0.05). In the severity-based subgroups, rs880633 appeared in 57.1% of non-severe and 61.9% of severe preeclamptics. Contrarily, the heterozygous form of rs7515776 polymorphism showed a significantly higher prevalence in the preeclamptic cohort (p<0.05), without distinctions in severity subgroups.

**CONCLUSION:**

The study suggests that the rs880633 polymorphism may serve a protective role against the development of preeclampsia, whereas the rs7515776 polymorphism may be associated with an elevated risk. Further research is warranted to elucidate the clinical implications of these findings.

## INTRODUCTION

One of the primary factors that contributes to maternal and perinatal mortality and morbidity during pregnancy is the presence of hypertensive disorders. These disorders manifest in approximately 5–10% of all pregnancies^1^. Hypertensive diseases during pregnancy can be classified into four categories: gestational hypertension, preeclampsia-eclampsia syndrome, chronic hypertension, and superimposed preeclampsia. According to the 2018 guidelines set forth by the International Society for the Study of Hypertension in Pregnancy (ISSHP), preeclampsia is defined as elevated systolic blood pressure (≥140 mmHg) and/or elevated diastolic blood pressure (≥90 mmHg), measured at least twice, at 4-h intervals, in otherwise healthy pregnant women. Moreover, preeclampsia is accompanied by one or more of the following new-onset conditions after the 20^th^ week of gestation: proteinuria, evidence of other maternal organ dysfunction, or uteroplacental dysfunction^2^. Preeclampsia is a multisystemic disorder characterized by hypertension, proteinuria, or end-organ damage, which may manifest as thrombocytopenia, renal dysfunction, hepatic dysfunction, pulmonary edema, or neurological or visual impairments. A critical role in the etiopathogenesis of both uteroplacental and systemic endothelial dysfunction is played by trophoblastic invasion deficiencies, which are essential for proper placental development. The etiology of preeclampsia is complex and multifactorial, involving oxidative stress, an elevated maternal immune response, ischemia, inflammation, and various immunological and environmental factors. Genetic predispositions also contribute to the etiology of preeclampsia^3-7^.

The *Chitinase 3-Like 1 (CHI3L1) gene* encodes for a glycoprotein, also known as YKL-40. This glycoprotein is produced by a myriad of human cells, including but not limited to macrophages, neutrophils, stem cells, bone cells, synoviocytes, chondrocytes, fibroblast-like cells, endothelial cells, vascular smooth muscle cells, hepatic stellate cells, mammary epithelial cells, and cancer cells. The CHI3L1 glycoprotein, secreted by activated neutrophils and macrophages, has been implicated in several crucial biological pathways, including angiogenesis, extracellular matrix reorganization, oncogenesis, and inflammation^8-10^. Gene polymorphisms represent single base-pair variations within genomic DNA that differentiate normal individuals within a population. In certain instances, these polymorphisms can significantly impact the function of the protein encoded by the gene or alter enzymatic activity. Such genetic polymorphisms are responsible for individual variances in susceptibility to illness and can significantly influence how individuals respond to medical treatment. The *CHI3L1 gene* exhibits multiple polymorphisms, and these genetic variations may influence both the incidence and prognosis of inflammatory and neoplastic diseases^11^. The objective of this study is to investigate any potential correlation between preeclampsia and polymorphisms in the *CHI3L1 gene* and specifically focus on the influence of polymorphisms in the *CHI3L1 gene* on the incidence and prognosis of preeclampsia.

## METHODS

### Ethics approval

This prospective case-control study obtained the necessary ethical approval from the local ethics committee (Approval Number 2020/220, dated 13.05.2020).

### Study design

The study was conducted between September 2020 and March 2021. It comprised 35 pregnant women diagnosed with preeclampsia who were admitted to our tertiary care center; these women constituted the study group (Group 1). The control group (Group 2) consisted of 40 healthy pregnant women. Preeclampsia was diagnosed based on elevated systolic blood pressure (≥140 mmHg) and/or diastolic blood pressure (≥90 mmHg), measured at least twice at 4-h intervals in otherwise healthy pregnant women, accompanied by one or more new-onset findings after the 20th week of gestation, such as proteinuria, evidence of other maternal organ dysfunction, or uteroplacental dysfunction^2^. Additionally, participants in Group 1 were further subdivided into severe and non-severe preeclamptic cases. The severity of the disease was assessed according to the recommendations of the American College of Obstetricians and Gynecologists^3^. Eligible participants for this study were females aged between 18 and 45 years with a singleton pregnancy. All patients were informed about the research objectives and procedures and signed an "Informed Voluntary Consent Form."

Exclusion criteria for the study included pregnant women with co-existing medical conditions such as diabetes mellitus, chronic hypertension, thromboembolism, thrombophilia, a history of liver or renal disease, structural or chromosomal fetal anomalies, multiple pregnancies, and gestational age less than 20 weeks. The control group comprised healthy pregnant women who did not present any additional features [e.g., placenta previa, intrauterine growth restriction (IUGR), and placental abruption] in their current or previous pregnancies.

Data, including age, gravidity, parity, blood pressure measurements, and complete blood count results [hemoglobin (Hb), white blood cell count (WBC), and platelets], were collected. Liver enzymes, specifically alanine aminotransferase (ALT) and aspartate aminotransferase (AST), were also recorded for all study participants.

### Genetic analysis

To analyze the *CHI3L1 gene* polymorphism in pregnant women, a 5 mL blood sample was collected from the antecubital brachial vein into an EDTA tube using a vacutainer. The blood samples were stored at +4°C until the completion of patient enrollment. The real-time polymerase chain reaction (PCR) technique was employed to study all the samples.

DNA isolation was performed using the DETAGEN Whole Blood DNA Isolation Kit (Detagen/Turkey). Following isolation, the densities (ng/μL) and absorption measurements (A260/280 ratio between 1.80 and 2.00) of the DNA samples were evaluated using a NanodropLite spectrophotometer (Thermo Scientific). The DETAGEN *CHI3L1 Gene* New Generation Sequencing Kit (Detagen/Turkey) was utilized for next-generation sequencing analysis. DNA purification was performed with AMPure XP Beads (Beckman Coulter, Indiana, USA), and sequencing was conducted on an Illumina MiSeq platform (Illumina, San Diego, California, USA).

The entire sequence of the *CHI3L1 gene* was screened in the serum of all participants. The distributions of both homozygous and heterozygous forms of the *CHI3L1 gene* polymorphisms were examined and compared between the two groups. Specifically, a total of 10 *CHI3L1 gene* polymorphisms were analyzed as follows:

c.433 A>G p.R145G (rs880633)

c.25+24 T>A (rs7515776)

c.-131 C>G (rs4950928)

c.56-19 T>C (rs1538372)

c.1092 T>C p.C364=(rs4950927)

c.55+32 C>T (rs111768615)

c.315-56 C>G (rs12410110)

c.257+5 G>A (rs201303588)

c.587+65 C>G (rs12409713)

c.894+9 G>T (no rs identification)

### Statistical analysis

All statistical analyses were performed using the SPSS 20 statistical software package (SPSS Inc., Chicago, IL). The distribution of homozygous and heterozygous forms of the polymorphism was statistically compared in terms of age, gravida and parity, blood pressure, hemogram, WBC, serum biochemistry, and *CHI3L1 gene* polymorphism in the pregnant women in Groups 1 and 2. Cases with severe and non-severe preeclampsia in Group 1 were compared statistically with the control group and among themselves. Categorical data were compared with chi-square analysis, the Mann-Whitney U test was used to compare two groups of numerical data, and the Kruskal-Wallis H test was used to compare three or more groups. A value of p<0.05 was considered statistically significant.

## RESULTS

No statistically significant differences were observed between the two groups regarding age, gestational weeks, gravida, and parity. However, significant differences were identified in the levels of systolic blood pressure (SBP) and diastolic blood pressure (DBP) between the control and preeclamptic groups (refer to Table 1).

**Table 1:** Demographic variables and blood pressure of the cases in both groups.

	Control (n=40)	Preeclampsia			p1	p2
		All PE (n=35)	Non-severe (n=14)	Severe (n=21)		
Age, years	28.7±5.17	29.65±7.48	29.42±8.05	29.80±7.27	0.528	0.811
Gravida	2 (1–6)	3 (1–6)	1.5 (1–5)	3 (1–6)	0.498	0.295
Parity	1 (0.0–3)	1 (0.0–4)	0 (0–4)	2 (0–3)	0.690	0.261
Gestational weeks	32	32	32.2	32	0.369	0.608
SBP	110 (100–120)	160 (140–190)	140 (140–150)	160 (150–190)	<0.001*	<0.001*
DBP	70 (60–70)	100 (70–150)	90 (70–110)	100 (90–150)	<0.001*	<0.001*

SBP: systolic blood pressure; DBP: systolic blood pressure; p1: comparison of control group and preeclampsia patients; p2: comparison of control group, severe, and non-severe preeclampsia.

* p<0.05 was accepted as statistically significant.

Among the nine analyzed *CHI3L1 gene* polymorphisms, only two showed statistically significant differences between the study groups: c.433 A>G p.R145G (rs880633) and c.25+24 T>A (rs7515776). For the remaining seven polymorphisms (rs4950928, rs1538372, rs4950927, rs111768615, rs12410110, rs201303588, rs12409713, and c.894+9 G>T), no significant differences were observed between the groups (p>0.05 for all).

Table 2 illustrates the distribution of homozygous and heterozygous forms of the c.433 A>G p.R145G (rs880633) polymorphism across groups. The prevalence of this polymorphism was 80% in the control group, 57.1% in the non-severe preeclampsia group, and 61.9% in the severe preeclampsia group. Notably, 8 out of 40 cases in the control group did not exhibit this polymorphism, representing 20% of the sample. The rate of homozygous polymorphisms was highest in severe preeclamptic cases (33.3%), compared with 17.5 and 21.4% in the control and non-severe groups, respectively. Conversely, the rate of heterozygous polymorphisms was higher in the control group (62.5% vs. 35.7 and 28.6%).

**Table 2:** Distribution of homozygous and heterozygous values of c.433 A>G p.R145G (rs880633) polymorphism between severe and non-severe preeclampsia group in preeclampsia and control group.

	Control (n=40)	Preeclampsia		p1	p2		OR 95%CI	p3	p4
		Non-severe (n=14)	Severe (n=21)						
No polymorphism	8 (20.0%)	6 (42.9%)	8 (38.1%)	0.026*	0.058		0.37 (0.13–1.04)	0.092	0.158
Polymorphism is present	32 (80.0%)	8 (57.1%)	13 (61.9%)						
Homozygous	7 (17.5%)	3 (21.4%)	7 (33.3%)						
Heterozygous	25 (62.5%)	5 (35.7%)	6 (28.6%)						

Data are given as n (%). p1=control and preeclampsia group with no polymorphism, comparison of homozygous and heterozygous; p2=control and preeclampsia group with no polymorphism comparing polymorphism exists; p3=control, severe, and non-severe preeclampsia group without polymorphism, comparison of homozygous and heterozygous; p4=control, comparison of severe and non-severe preeclampsia group with no polymorphism with polymorphism exists.

* p<0.05 was accepted as statistically significant.

Regarding the c.25+24 T>A (rs7515776) polymorphism, its prevalence was lower in healthy pregnancies (65% vs. 35%), as depicted in Table 3. Within the preeclampsia cohort, the rates of this polymorphism were 57.1% for non-severe cases and 38.1% for severe cases. No instances of homozygous polymorphism were observed in the preeclamptic patients.

**Table 3:** Distribution of homozygous and heterozygous values of c.25+24T>A(rs7515776) polymorphism between severe and non-severe preeclampsia group in preeclampsia and control group.

	Control (n=40)	Preeclampsia			p1	p2	OR 95%CI	p3	p4
		All PE (n=35)	Non-severe (n=14)	Severe (n=21)					
No polymorphism	26 (65.0)	19 (54.3)	6 (42.9)	13 (61.9)	0.046*	0.345	1.56 (0.61–3.96)	0.109	0.339
Polymorphism is present	14 (35.0)	16 (45.7)	8 (57.1)	8 (38.1)					
Homozygous	4 (10.0)	0 (0.0)	0 (0.0)	0 (0.0)					
Heterozygous	10 (25.0)	16 (45.7)	8 (57.1)	8 (38.1)					

Data are given as n (%). p1=control and preeclampsia group with no polymorphism, comparison of homozygous and heterozygous; p2=comparison of control and preeclampsia group with no polymorphism with polymorphism exists; p3=control, severe, and non-severe preeclampsia group with no polymorphism, comparison of homozygous and heterozygous; p4=control, comparison of severe and non-severe preeclampsia group with no polymorphism with polymorphism exists.

* p<0.05 was accepted as statistically significant.

## DISCUSSION

The primary finding of our study suggests that the polymorphism rs880633 in the *Chitinase-3-like protein 1 (CHI3L1) gene* may serve as a protective factor against preeclampsia. This is a pioneering study, as this is the first to explore the relationship between *CHI3L1 gene* polymorphisms and preeclampsia. Preeclampsia is a significant cause of maternal morbidity and mortality, and currently, no standard screening tests are available for its diagnosis, as indicated by previous studies^12,13^.

*CHI3L1*, which is a biomarker involved in inflammation and tissue remodeling, plays a supportive role in angiogenesis, antiapoptosis, and cell proliferation^8-11^. Dina Nada et al. investigated the relationship of circulating YKL-40 levels and CHI3L1 variants with the risk of progression of scoliosis to spinal deformity in adolescent idiopathic scoliosis. It has been shown that the rs880633 polymorphism of the *CHI3L1 gene* is positively correlated with high YKL-40 levels and is protective against spinal deformity^14^. Huang et al. examined the polymorphisms of the *CHI3L1 gene* in patients with hepatocellular cancer and found a high rate of CHI3L1rs880633 polymorphism in patients with hepatocellular cancer^15^. CHI3L1 has been identified as a promoter of angiogenesis in neoplasms and has been shown to play a role in the activation of the mitogen-activated protein kinase/extracellular signal-regulated kinase pathway in endothelial cells^16,17^. CHI3L1 has been shown to modulate vascular endothelial cell morphology and stimulate migration. CHI3L1 plays a role in tumor angiogenesis^18^. In our study, CHI3L1rs880633 polymorphism was detected at a higher rate in the control group. This aligns with our observation that the rs880633 polymorphism was more prevalent in our control group, suggesting that it may confer protective effects, potentially mediated through angiogenesis.

El-Fattah et al. investigated the rs4950928 polymorphism of the *CHI3L1 gene* in patients with colorectal cancer (CRC). They could not find a significant relationship between CHI3L1 rs4950928 polymorphism and CRC^19^. Dai et al. investigated whether CHI3L1 polymorphisms and plasma level of protein are associated with Alzheimer's disease. This study showed that the CG+GG genotype of rs4950928 C>G is a protective factor for Alzheimer's disease and reduces the severity of Alzheimer's disease^20^. The CHI3L1 rs4950928 genotype was investigated in patients with glioblastoma (GBM), and no significant correlation was found between glioblastoma and the CHI3L1 rs4950928 genotype^20,21^. Our study found no significant relationship between *CHI3L1 gene* rs4950928 polymorphism and preeclampsia.

YKL-40 levels with CHI3L1 rs 1538372 polymorphism and its relationship to lung function were investigated. Tsai et al. found decreased lung functions in CHI3L1 rs1538372 CC carriers^22^. In our study, no significant relationship was found between the rs1538372 polymorphism of the *CHI3L1 gene* and preeclampsia.

The effects of rs7515776 polymorphism of *the CHI3L1 gene* and YKL-40 levels on the course of the disease were investigated in sarcoidosis patients^23^. There was no correlation between rs7515776 polymorphism and serum YKL-40 levels in sarcoidosis patients. This study observed that the rs7515776 polymorphism showed a higher heterozygosity rate in the preeclampsia group. However, the absence of homozygosity prevents us from attributing definitive clinical significance to this finding.

There are no studies in the literature associated with rs4950927, rs111768615, rs12410110, rs201303588, rs12409713, and c.894+9 G>T (no rs) polymorphisms of the *CHI3L1 gene*. In this study, a significant correlation was not found between rs4950927, rs111768615, rs12410110, rs201303588, rs12409713, and c.894+9 G>T (no rs) polymorphisms of the *CHI3L1 gene* and preeclampsia.

One limitation of our research is the small sample size and the absence of detailed birth records and perinatal outcomes. These factors could have provided a more nuanced understanding of the clinical implications of these polymorphisms.

In summary, our study provides a promising direction for future research, suggesting that the rs880633 polymorphism in the *CHI3L1 gene* could act as a protective factor against preeclampsia. More comprehensive studies with larger sample sizes and a more extensive range of clinical data are required to confirm these initial findings.
